# Easy to process, hard to control: Transient and sustained processing fluency impairs cognitive control adjustments to conflict

**DOI:** 10.1177/17470218231159787

**Published:** 2023-03-23

**Authors:** Gonçalo A Oliveira, Miguel Remondes, Teresa Garcia-Marques

**Affiliations:** 1 ISPA-William James Center for Research, Lisboa, Portugal; 2 Instituto de Medicina Molecular João Lobo Antunes, Universidade de Lisboa, Lisboa, Portugal; 3 ISPA—Instituto Universitário, Lisboa, Portugal

**Keywords:** Control, fluency, congruence, conflict

## Abstract

Recent research suggests that the cognitive monitoring system of control could be using negative affective cues intrinsic to changes in information processing to initiate top-down regulatory mechanisms. Here, we propose that positive feelings of ease-of-processing could be picked up by the monitoring system as a cue indicating that control is not necessary, leading to maladaptive control adjustments. We simultaneously target control adjustments driven by task context and on a trial-by-trial level, macro-, and micro-adjustments. This hypothesis was tested using a Stroop-like task comprised trials varying in congruence and perceptual fluency. A pseudo randomisation procedure within different proportion of congruence conditions was used to maximise discrepancy and fluency effects. Results suggest that in a mostly congruent context participants committed more fast errors when incongruent trials were easy-to-read. Moreover, within the mostly incongruent condition, we also found more errors on incongruent trials after experiencing the facilitation effect of repeated congruent trials. These results suggest that transient and sustained feelings of processing fluency can downregulate control mechanisms, leading to failed adaptive adjustments to conflict.

## Introduction

Cognitive control has been described as a set of mechanisms that allow goal pursuit in the presence of distractors or strong dominant responses (Botvinick et al., 2001). According to the control monitoring theory (CMT), control is activated when the monitoring system detects the occurrence of conflict, resulting from a cross-talk between parallel information processing pathways (Botvinick, 2007; Botvinick et al., 2001). However, research on cognitive control has accumulated evidence that the activation of top-down regulatory mechanisms can also be elicited in the absence of conflict through, for instance, fluency manipulations on perception (Dreisbach & Fischer, 2011) or task context (Dreisbach et al., 2018). Interestingly, cognitive conflict and disfluency are experienced as affective experiences. It has been suggested that cognitive conflict is intrinsically aversive and that this negative affect could enhance the need-for signal, mobilising control mechanisms (Dreisbach & Fischer, 2015; Inzlicht et al., 2015). Similarly, whereas fluency has a positive tone, disfluency seems to be more tightly linked to negative affect (Garcia-Marques et al., 2016; Topolinski & Strack, 2015; Winkielman et al., 2003) and its overall effects on information processing are consistent with the activation of control (Alter et al., 2007; Garcia-Marques et al., 2013). A recent experiment has found that changes in perceptual fluency–disfluency and congruence–incongruence lead to additive adjustments in control mechanisms (Oliveira et al., 2022). These findings suggest that although perceptual disfluency and incongruence have different sources, they can be integrated in the cognitive system as a general experience of disfluent information processing, matching the predictions of Alter and Oppenheimer (2009). Moreover, in line with previous research (e.g., Dreisbach et al., 2018; Dreisbach & Fischer, 2011), it brings into attention the possibility that the monitoring system of control could be using changes in ease-of-processing as a cue to activate the control mechanisms and not strictly instances of conflict as theorised (Botvinick, 2007; Botvinick et al., 2001).

If the monitoring system is indeed using the experience of disfluent processing as a cue to initiate top-down regulatory control, then any other source eliciting an experience of ease-of-processing could interfere with the signal. Consequently, ease-of-processing may impair the adaptive activation of control mechanisms by signalling that control is not required. Matching this hypothesis, researchers have induced incidental ease-of-processing in cognitive demanding tasks, showing that it leads participants to experienced positive affect and to rely more on heuristic/automatic responses (Claypool et al., 2015; Volz et al., 2010). Moreover, cognitive illusions are less detected when presented in a perceptual fluent manner (Song & Schwarz, 2008) and congruent trials within a Simon task have been shown to relax control mechanisms and increase the costs of sequential control adaptations, possibly because they were experienced as fluent processing (Berger et al., 2019).

Our present aim is now to investigate the mechanisms through which ease-of-processing affects control adjustments. Using the nomenclature of Ridderinkhof (2002), we will simultaneously target macro-adjustments, which refer to the changes in control elicited by the task context (e.g., proportion of incongruent trials within the task), and micro-adjustments driven by trial-by-trial contingencies. To manipulate ease-of-processing, we will manipulate perceptual fluency by presenting the stimuli in easy and hard-to-read fonts (Oliveira et al., 2022; Song & Schwarz, 2008). We expect control activation for stimuli presented in a disfluent manner (Dreisbach & Fischer, 2011; Oliveira et al., 2022; Song & Schwarz, 2008). We hypothesise that the experience of ease-of-processing will potentiate automatic responses in incongruent trials before inhibitory control is sufficiently activated. Moreover, since the efficiency of suppression mechanisms builds up with time spent within a trial (Wylie et al., 2009), we predict that participants will commit more errors on fast responses to easy-to-read trials, reflecting the unchecked activation of the automatic response. It is expected that the response activation effect will be attenuated when trials are hard-to-read. Furthermore, this effect should dissipate independently of perceptual fluency on longer response times (RTs) due to a sufficient activation of inhibitory control. To properly test this hypothesis, we will use distributional analysis of accuracy and RT (delta-plots; Ridderinkhof, 2002), that allow the plotting of experimental condition differences over RT.

Critically, for control mechanisms to be rendered ineffective, task context must be considered. Therefore, to bias the process, the task context must guarantee that cognitive control will be sufficiently relaxed. The dual mechanisms of control framework propose that cognitive control operates via two qualitatively distinct modalities that are sensitive to contextual and inter-individual variables (Braver, 2012; Braver et al., 2007; Tang et al., 2022). More recent research suggests that these modalities could reflect independent control mechanisms (Gonthier et al., 2016). Theoretically, reactive control is activated only as needed, following the detection of a high interference event and is thought to rapidly decay after the resolution of interference (Braver, 2012; Braver et al., 2007). Critically, given the transient nature of reactive control, the system is more easily influenced by bottom-up inputs and shows a greater dependence on the detection of trigger events (Braver et al., 2007). On the other hand, proactive control reflects the anticipatory and/or sustained activation of control. Under this modality, the active maintenance of task goals functions as a source of top-down bias, facilitating the processing of relevant upcoming events (Braver, 2012; Braver et al., 2007).

Previous research varied the proportion of items in the task to manipulate the control demands (concomitantly the different modalities of control) and found a reduction of Stroop interference when tasks comprised predominantly incongruent items (e.g., De Pisapia & Braver, 2006; West & Bailey, 2012). In our experiment, this manipulation will be performed between-subjects. We predict that the effect of ease-of-processing on automatic response activation will be restricted to the mostly congruent condition in which control mechanisms are expected to only be activated following the detection of conflict trials (Braver, 2012; Braver et al., 2007). On the other hand, it should be harder to detect evidence of this response capture effect on the mostly incongruent condition. In this condition, control is expected to be sustained throughout the task, and therefore, response inhibition mechanisms should already be proactively activated at the beginning of the trial, matching the predictions of the dual mechanisms of control framework (Braver, 2012; Braver et al., 2007).

For a more complete micro-level analysis, we will target specific trial sequences. This will allow studying the impact of unexpected processing fluency on control mechanisms. Previous research has shown that our processing experiences are context relative. Fluency is thought to be a relative experience that signals the level of discrepancy between the expected and the actual experience of information processing (Whittlesea & Williams, 1998, 2001). Hence, the same fluent stimulus can activate a higher experience of ease-of-processing when preceded by a disfluent stimulus, than when presented after other fluent stimuli. To bias control mechanisms, we will use a pseudo randomisation procedure to ensure that discrepant trials (incongruent in mostly congruent, congruent in mostly incongruent) are presented in single trials or in repeated presentations, while balancing all transitions in perceptual fluency. This procedure should maximise the feeling of discrepancy within the task and induce an unexpected feeling of processing fluency in the congruent trials of the mostly incongruent condition, relaxing the upregulated control mechanisms (Berger et al., 2019) and impacting the following incongruent trial.

## Methods

### Power analysis

On this experiment, the a priori power analysis was performed by iteration using PANGEA (Westfall, 2016) and complemented with a databased and SESOI (estimates reduced by 15%) power simulation using the R package mixedpower (Kumle et al., 2021) on the data from Oliveira et al. (2022), which used the same task and set of stimuli as the experiment reported here. On all power analyses, the total number of trials in the task (384) and the number of replicates by condition (minimum of 48 trials) was fixed and predetermined, so that, the delta-plot analysis could be performed, leaving only to vary the estimates for the required number of participants. We have used the effect sizes on accuracy and RT reported by West and Bailey (2012) on their counting Stroop experiment as estimates for the main effect of congruence (accuracy: η^2^_*p*_ = 70; RT: η^2^_*p*_ = 92), proportion of congruence (accuracy: η^2^_*p*_ = 35; RT: η^2^_*p*_ = .14) and the congruence × proportion of congruence interaction (accuracy: η^2^_*p*_ = 34, RT: η^2^_*p*_ = .51). For fluency effects, we have used Dreisbach and Fischer’s (2011) effect size (η^2^_*p*_ = .38) as an estimate. Finally, the effect size for the relevant differences in the congruence × fluency × proportion of congruence three-way interaction (*d* *=* .33) was derived from the work of Oliveira et al. (2022). Overall, the power analyses indicate that statistical power is above .80 for sample sizes of 80 participants or more.

### Participants and design

Ninety-two participants (80 women, 12 men; age: *M* = 22.03, *SD* *=* 4.95) were recruited through the ISPA-Instituto Universitário subject pool and received one course credit for their participation in this experiment. All the procedures described here were approved by ISPA Ethics Committee. Participants were randomly assigned to the experimental conditions in a mixed design of 2 (congruence: congruent and incongruent) × 2 (legibility: easy-to-read and hard-to-read) × 2 (proportion of congruence: mostly congruent and mostly incongruent). Congruence and legibility were manipulated within-subjects and proportion of congruence between-subjects.

### Task and procedure

Participants performed a version of the counting Stroop task (West & Bailey, 2012) that also manipulates perceptual fluency (Oliveira et al., 2022). In this task, subjects are instructed to identify the number of digits presented on screen for congruent (identity of the digit matches the number of digits—e.g., 22) and incongruent trials (identity of the digit does not match the number of digits—e.g., 222). Font legibility (20 easy-to-read and 20 hard-to-read fonts; see Table S1 of the Supplementary material) of congruent and incongruent stimulus was manipulated to elicit different levels of perceptual fluency. Digits presented in Arial size 18 were used as a reference, to control for font-dependent variations in character size.

The experimental task comprised one training block and two testing blocks. Task instructions stressed that a correct response should be given as fast as possible. Participants were first asked to perform a training block with 30 easy-to-read trials (15 congruent and 15 incongruent). Response feedback (presentation of the word “correct” or “incorrect” for 1,000 ms) was only given on this phase of experiment. After completing the training block, participants initiated the testing phase. In this phase, participants responded to a total of 384 trials (two blocks of 192 trials). Depending on their randomly assigned experimental condition, participants either performed the task with mostly congruent (congruent: 144 easy-to-read and 144 hard-to-read; incongruent: 48 easy-to-read and 48 hard-to-read) or with mostly incongruent stimuli (congruent: 48 easy-to-read and 48 hard-to-read; incongruent: 144 easy-to-read and 144 hard-to-read). It is important to note that the proportion of easy-to-read/hard-to-read trials was maintained in both proportion of congruence conditions.

To study the effect of discrepancy within the proportion of congruence conditions, we established sequences of interest comprising the discrepant trials (e.g., incongruent trials in the mostly congruent condition, congruent trials within the mostly incongruent condition) and the frequent trial presented before and after the discrepant trials. Discrepant trials were presented in an equal number of single presentation or presented in two consecutive trials (32 each). All perceptual fluency transitions within these sequences of interest were counterbalanced. These sequences of interest were then embedded within the proportion of congruence conditions and pseudorandomised with the remaining more frequent trials using Mix (Van Casteren & Davis, 2006). The pseudo-randomisation procedure set the following rules of stimulus presentation: (a) sequences of interest should be separated by at least one more frequent type of trial; (b) each testing block should have the same number and type of sequence of interest; and (c) the same font could not be presented in consecutive trials, to minimise undesired fluency effects elicited by font repetition. This process was repeated to generate six pseudorandomised lists of stimulus presentation for each proportion of congruence condition, which were then implemented in the computerised task.

The beginning of each block was signalled by a black fixation cross that was displayed on the centre of the screen for 1,000 ms. The stimuli were presented in black a white background and remained on the centre of the screen until a response was given (response keys: 1–4). The response to stimulus interval was set to 500 ms. Between each block, participants could rest for a maximum duration of 15 s or resume the task at any point by pressing a key. The computerised task was deployed using E-Prime 2.0 (Psychology Software Tools, Pittsburgh, PA).

### Data processing and statistical analysis

First, we have used the overall proportion of correct responses above 75% as a proxy of compliance with the task goals and to control for fatigue effects. Four participants were excluded from further analysis based on these criteria. Trial RT with values below 300 ms and above 3,000 ms was replaced by missing values. RTs were then log-transformed (base 10) and checked for outliers ± 3 standard deviations from the mean. Outlier observations were replaced by missing values.

For the accuracy and RT delta-plots, we have followed the procedure described by Wylie et al. (2009). To compute the accuracy delta-plot, trial RTs of correct and incorrect responses were rank ordered and split into five bins with an equal number of trials. The accuracy rate of each experimental condition was then computed for each of the bins. Finally, for each bin, we created an index by subtracting the accuracy of incongruent and congruent trials. In the accuracy delta-plot analysis, we will focus on the accuracy of the first bin (faster responses) and on the slope connecting the first two bins. These measures are thought to reflect rapid response activation processes that escape response inhibition (Wylie et al., 2009). For RT delta-plots, we have only used trials associated with a correct response. The RTs of these trials were rank-ordered and segmented in five bins with an equal number of trials. An index of interference was then calculated for each bin by subtracting the bin mean of incongruent and congruent trials. Contrasting with the analysis for accuracy, on the RT delta-plot, we will focus on the last bin (slower responses) and on the slope connecting the last two bins. Since suppression builds over time, these measures should reflect condition differences in effective response inhibition (Wylie et al., 2009). It is important to note that on delta-plots, bins are formed based on ranked trial RT, irrespective of the moment in which they were presented to the participant.

Statistical analysis was performed in R (R Core Team, 2021) using generalised linear mixed models on the accuracy data and linear mixed models for RT based variables using the package lme4 (Bates et al., 2015) and lmerTest (Kuznetsova et al., 2017). Mixed models with difference random-effect structures were computed and, after successful convergence, compared using a likelihood-ratio test to select the final model presented in this manuscript. Random intercepts for subjects and for targets (e.g., stimuli) were entered in all models, except on delta-plots, which combined the measures of multiple targets. The overall RT model included random slopes for congruence within subjects and both delta-plot models included random slopes for legibility within subjects. Finally, the RT model for discrepant trials included random slopes for trial repetition within subjects. Fixed effects were always congruence, legibility, proportion of congruence, and their interactions. On the conditional accuracy function (CAF) and RT delta-plot models, a fixed effect for bins was also added. Estimated marginal means were computed using emmeans (Lenth, 2020). For the analysis of discrepant trial repetition and their impact on the following trial, separate models were computed for the mostly congruent and mostly incongruent condition to avoid confounds. Contrast analysis within the models was performed following Rosenthal and Rosnow (1985) guidelines.

## Results

### Accuracy and reaction times

**Figure 1.**
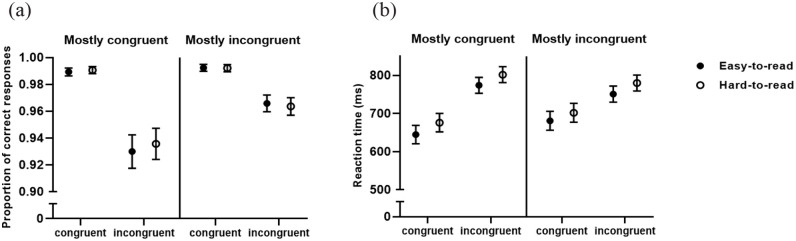
Proportion of correct responses (a) and trial reaction times (b) for easy and hard-to-read trials by congruence within each proportion of congruence conditions (*M* ± *SE*).

As expected, the statistical analysis indicates that participants were overall faster and more accurate on congruent trials (see Table 1; Figure 1a and b). In addition, and also replicating previous research, the congruence × proportion of congruence condition interaction was significant, suggesting that the Stroop effects on both accuracy and RT was higher on the mostly congruent, compared with the mostly incongruent condition (accuracy: *z* *=* 2.35, *p* *=* .019, *odds ratio* = 0.18; *RT: t*(87.2) = 8.29, *p* *<* .001, *d* *=* 0.31). For accuracy, we have also found a main effect for the proportion of congruent trials, suggesting higher accuracy for participants in the mostly incongruent condition compared with the mostly congruent condition. No other effect was significant.

**Table 1.** Fixed-effect statistics of the linear mixed models for accuracy and reaction times.

Effect	Accuracy			Reaction times		
	*df*	χ²	*p*-value	*df*	*F*-value	*p*-value
Congruence	1	19.21	<.001***	1, 15	27.18	<.001***
Legibility	1	.12	.724	1, 32,059	119.48	<.001***
Proportion of congruence	1	6.62	.010**	1, 86	.07	.793
Congruence × legibility	1	.04	.835	1, 32,059	.08	.774
Congruence × proportion of congruence	1	5.02	.025*	1, 87	68.69	<.001***
Legibility × proportion of congruence	1	.81	.368	1, 32,058	.32	.566
Congruence × legibility × proportion of congruence	1	<.01	.971	1, 32,058	2.81	.093

*df*: degrees of freedom (numerator, denominator).

* Significant effect for *p* ⩽ .05; **Significant effect for *p* ⩽ .01; ***Significant effect for *p* ⩽ .001.

Stimulus legibility effects were only detected for RT. The main effect of legibility suggests that it promoted smaller RTs on easy-to-read trials. Relevant to our goals, we have found a marginal three-way interaction of congruence × legibility × proportion of congruence condition. The difference between mostly congruent and mostly incongruent for the congruence effect is maintained on easy-to-read (*t*(187.7) = 7.79, *p* *<* .001, *d* *=* 0.36) and hard-to-read trials (*t*(187.1) = 5.90, *p* < .001, *d* *=* 0.27). Although the magnitude of the effect was smaller on hard-to-read trials, the contrast comparing the differences in the Stroop effect on easy-to-read and hard-to-read trials between proportion of congruence conditions was only marginal (*t*(32,058.3) = 1.68, *p* *=* .09, *d* *=* 0.09).

### Accuracy delta-plot

**Figure 2.**
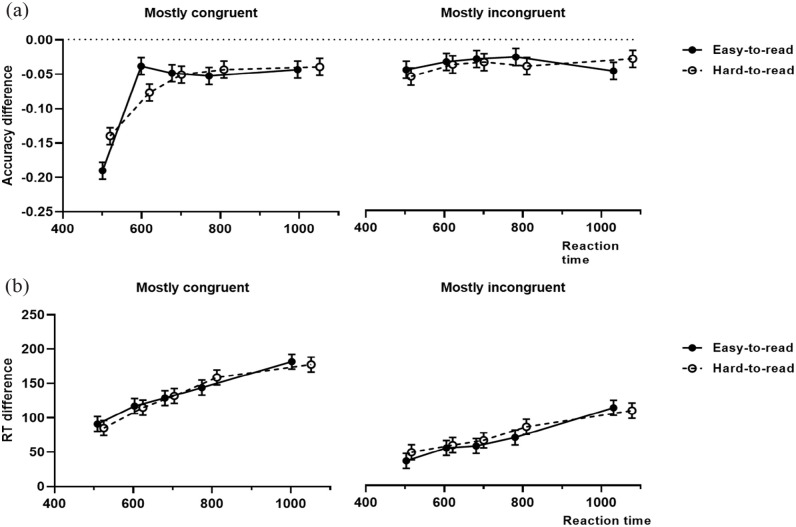
Delta-plot for accuracy (a) and reaction time (b) for easy and hard-to-read trials within each proportion of congruence condition (*M* ± *SE*).

Here, we aimed to investigate how the interference effect on accuracy developed in function of the participant’s RT. We have found a three-way interaction between the accuracy index × bins × proportion of congruence (*F*(4, 774) = 2.53, *p* *=* .039; Figure 2a). We have used a trend analysis to investigate how the Stroop effect on accuracy of easy and hard-to-read trials varies over binned RT in the mostly congruent and mostly incongruent conditions. Significant linear and quadratic components for the Stroop effect on the accuracy of easy (linear: *t*(774) = 7.90, *p* *<* .001, *d* *=* 3.72; quadratic: *t*(774) = 6.66, *p* *<* .001, *d* *=* 3.71) and hard-to-read trials (linear: *t*(774) = 6.62, *p* *<* .001, *d* *=* 3.12; quadratic: *t*(774) = 3.28, *p* *=* .001, *d* *=* 1.82) were detected on the mostly congruent condition. However, only the quadratic trend for easy-to-read trials was significantly different from hard-to-read trials in this proportion of congruence condition (linear: *t*(774) = .90, *p* *=* .366, *d* *=* 0.60; quadratic: *t*(774) = 2.39, *p* *=* .017, *d* *=* 1.89). On the mostly incongruent condition, we have not found significant trends on easy (linear: *t*(774) = 0.11, *p* *=* .912, *d* *=* 0.05; quadratic: *t*(774) = 1.52, *p* *=* .126, *d* *=* 0.87) or hard-to-read trials (linear: *t*(774) = 1.36, *p* *=* .174, *d* *=* 0.66; quadratic: *t*(774) = .54, *p* *=* .590, *d* *=* 0.30).

To look for evidence of response capture, we have conducted a planned contrast analysis on the accuracy rate of the first bin and the slope connecting the first two bins of the accuracy delta-plot. We have found that participants in the mostly congruent condition committed more fast errors when responding to easy-to-read trials, than when trials were hard-to-read (*t*(774) = 3.17, *p* *=* *.002*, *d* *=* 0.67). Moreover, in the mostly congruent condition, we have also found a significantly steeper positive slope connecting the first two bins of the Stroop effect on easy-to-read, indicating a stronger effect of response capture (*t*(774) = 2.90, *p* *=* .005, *d* *=* 4.52). These effects were not detected on the mostly incongruent condition (Bin 1 contrast: *t*(774) = .60, *p* *=* .550, *d* *=* 0.12; slope contrast: *t*(86) = .22, *p* *=* .828, *d* *=* 0.35). The full fixed-effects for the accuracy delta-plot and slopes models are reported on Table S2–S3 of the Supplementary material.

### Reaction time delta-plot

The RT delta-plot analysis will focus on how the interference effect on RT varies depending on the participant’s binned RT (Figure 2b). As expected, we have found a significant main effect for the proportion of congruence suggesting higher interference on the mostly congruent condition compared with the mostly incongruent condition. We have also found a main effect for bins indicating that interference increases over RT. Consistent with this evidence, we have found significant linear trends for interference on easy and hard-to-read trials on both proportion of congruence conditions (mostly congruent, easy-to-read: *t*(676) = 7.22, *p* *<* .001, *d* *=* 3.48; hard-to-read: *t*(676) = 8.00, *p* *<* .001, *d* *=* 3.82; mostly incongruent, easy-to-read: *t*(676) = 5.92, *p* *<* .001, *d* *=* 2.85; hard-to-read: *t*(676) = 5.14, *p* *<* .001, *d* *=* 2.47). However, no significant differences were detected between the linear trends of easy and hard-to-read trials on the mostly congruent (*t*(676) = 0.50, *p* *=* .619, *d* *=* 0.33) or mostly incongruent condition (*t*(676) = 0.55, *p* *=* .582, *d* *=* 0.38).

To investigate differences in response inhibition, we conducted a planned contrast analysis on the last bin of the RT delta-plot (Figure 2b) and on the slope connecting the last two bins. We did not find significant differences in response inhibition between easy and hard-to-read trials for the mostly congruent (Bin 5 contrast: *t*(513) = 0.30, *p* *=* .761, *d* *=* 0.07; slope contrast: *t*(85.1) = 0.41, *p* *=* .685, *d* *=* 0.09) or the mostly incongruent proportion of congruence conditions (Bin 5 contrast: *t*(511) = 0.29, *p* *=* .767, *d* *=* 0.07; slope contrast: *t*(84.2) = 0.037, *p* *=* .713, *d* *=* 0.08). We present the RT delta-plot and slopes models statistics on Table S2–S3 of the Supplementary material.

### Accuracy, reaction time, and impact of discrepant trials

**Figure 3.**
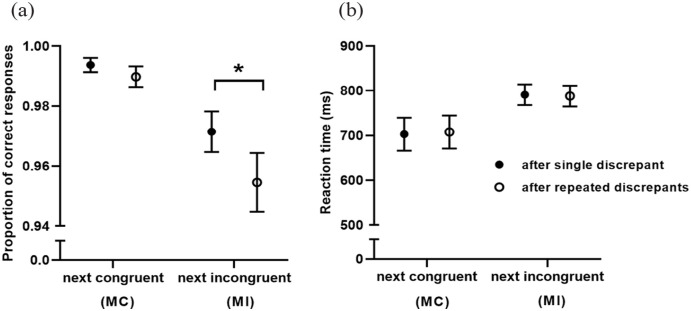
Impact of discrepant trials on accuracy (a) and reaction time (b) of the following trial for each proportion of congruence condition (*M* ± *SE*). MC: mostly congruent; MI: mostly incongruent. *Significant difference for *p* ⩽ .05.

This set of analyses investigated the participant’s accuracy and RT on discrepant trials (e.g., less frequent) and what were the repercussions of single or repeated exposure of these trials on the next more common type of trial. Participants were significantly more accurate and faster on the second exposure to congruent trials on the mostly incongruent condition (see Table 2 for fixed-effects statistics; descriptive statistics are presented on Table S4 of the Supplementary material). Moreover, after experiencing these repeated congruent trials, participants in this condition committed more errors on the following incongruent trial, compared with instances in which a single congruent trial was presented (Figure 3a; Table 2). No significant differences on the following trial were detected for RT (Figure 3b; Table 2). Moreover, no main effect of legibility or interaction between legibility and repetition was detected for accuracy or RT. On the mostly congruent condition, participants were slower on repeated incongruent, but we did not find effects on accuracy. Furthermore, the repetition of incongruent trials did not seem to affect the accuracy or the RT of the following congruent trial (Figure 3a and b; see Table S5 for descriptive statistics). However, we have found that participants were faster on the next congruent trial after experiencing incongruence, when both trials were easy-to-read or when there was a transition from hard-to-read to easy-to-read (*t(*2,066) = 3.90, *p* *<* .001, *d* *=* 0.34. No interaction between repetition and legibility was detected on accuracy or RT.

**Table 2.** Fixed-effect statistics of the linear mixed models for the accuracy and reaction times of discrepant trials within each proportion of congruence condition.

Proportion of congruence	Model	Effect	Accuracy			Reaction time		
			*df*	χ²	*p*-value	*df*	*F*-value	*p*-value
Mostly congruent	Discrepant trial	Repetition	1	.03	.870	1, 45.22	7.38	.009**
	Following trial	Repetition	1	2.05	.152	1, 2,066.2	< 1	.988
		Legibility	3	1.34	.719	3, 2,066	5.17	.001**
		Repetition × legibility	3	.44	.931	3, 2,066.1	1.41	.239
Mostly incongruent	Discrepant trial	Repetition	1	4.12	.042*	1, 41.77	6.51	.014*
	Following trial	Repetition	1	6.91	.008**	1, 1,921.7	< 1	.973
		Legibility	3	.55	.907	3, 1,920.7	2.04	.105
		Repetition × legibility	3	4.04	.257	3, 1,919.8	1.62	.182

*df*: degrees of freedom (numerator, denominator).

* Significant effect for *p* ⩽ .05; **Significant effect for *p* ⩽ .01.

## Discussion

In this experiment, we tested the hypothesis that different levels of ease-of-processing (with the same affective tone) are integrated in the cognitive system, so that, the experience of ease-of-processing becomes a cue that indicates that the activation of control is not required. As such, incidental manipulations of fluency of processing are expected to impair the participants’ performance on a control-demanding task, such as the counting Stroop. In our experiment, we expected to better detect this impairment (participants being more susceptible to processing fluency-driven errors), on easy-to-read trials embedded in conditions known to relax the mechanisms of control due to a high frequency of congruent trials (mostly congruent condition; Braver, 2012). Moreover, since inhibitory mechanisms of control are thought to increase effectiveness when more time is spent within a trial (Ridderinkhof, 2002; Wylie et al., 2009), we expect that the misleading effect of ease-of-processing will be mostly reflected on trials with a faster RT. Our results are consistent with these predictions. We have found that participants specifically committed more fast errors (Bin 1) on easy-to-read trials of the mostly congruent condition (see accuracy delta-plot on Figure 2a). Furthermore, we have also found significant differences in the slopes connecting the first two bins of easy and hard-to-read trials, which can be interpreted as further evidence that response activation was stronger on easy-to-read trials in this proportion of congruence context (Ridderinkhof et al., 2004).

Importantly, task accuracy and RT results indicate that the manipulation of proportion of congruence and of perceptual fluency were successful in creating the experimental conditions to address, ultimately supporting our initial predictions. We have found a larger Stroop effect on accuracy and RT on the mostly congruent condition compared with mostly incongruent, replicating previous research (De Pisapia & Braver, 2006; West & Bailey, 2012). These findings indicate that control mechanisms in the mostly congruent condition were likely more relaxed, being activated on a need-to basis when rare instances of incongruence were detected (Braver, 2012; Braver et al., 2007). Moreover, participants were slower when the stimulus was hard-to-read, which is consistent with a more disfluent type of processing in these trials (Dreisbach & Fischer, 2011; Song & Schwarz, 2008). Notably, the Stroop effect is maintained on both levels of perceptual fluency (although marginally smaller on hard-to-read trials), as indicated by the contrast analysis on the three-way interaction between congruence, legibility, and proportion of congruence, replicating previous findings (Oliveira et al., 2022). Although these fluency signals originate from different sources, they may be integrated in the cognitive system as a general feeling of processing fluency (Alter & Oppenheimer, 2009) and elicit additive control adjustments (Oliveira et al., 2022).

According to the activation–suppression hypothesis (Ridderinkhof, 2002), activation of automatic responses, that are not compatible with task goals, can occur on a direct route and lead to a motor response, before the threshold of a more deliberate correct response can be reached. In the current experiment, it is likely that the errors detected on fast responses to easy-to-read trials in the mostly congruent condition result from the activation of the more automatic number reading response, which was amplified by feelings of processing fluency. On the other hand, when trials were perceptually hard-to-read, the automatic response may have been sufficiently slowed down, so that, it could be more effectively targeted by inhibitory mechanisms (Ridderinkhof, 2002; Ridderinkhof et al., 2004), which is consistent with other research on fluency and control (Dreisbach et al., 2018; Dreisbach & Fischer, 2011; Oliveira et al., 2022).

Interestingly, the trend analysis of the delta-plot for accuracy on the mostly congruent condition indicates that performance on easy-to-read trials improved at a faster rate, than on hard-to-read to trials as indicated by the significant differences in quadratic trends. These results suggest that the effects described above are not constant over time, and that the observed slight impairment in suppressing the response activation on easy-to-read trials is quickly followed by a marked improvement, suggesting efficient adaptation to processing fluency. These findings seemingly contradict what has been described for the effects of fluency on information processing, specifically that it elicits more automatic types of processing (Alter et al., 2007; Garcia-Marques et al., 2013), impairing the detection of cognitive illusions (Song & Schwarz, 2008). An explanation for these findings can be found in the activation–suppression hypothesis (Ridderinkhof, 2002). This model proposes that when the decisional processes supporting the deliberate response are relatively fast, this response can be given before the automatic response reaches the threshold of an overt response (Ridderinkhof, 2002). Our results are consistent with this prediction and were interpreted as evidence that the perceptual fluency on easy-to-read trials increased the activation of both the automatic and the deliberate responses, causing more errors when responses are the fastest, but allowing the deliberate response to quickly take over with more time spent on the trial. It is important to note that in the mostly incongruent condition, we did not detect any difference between easy and hard-to-read trials. In fact, the trend analysis indicates that in this condition, accuracy did not improve with RT. The high proportion of incongruent trials in mostly incongruent is thought to lead to a more sustained activation of control, facilitating the resolution of conflict (Braver, 2012; Braver et al., 2007). This upregulation of control mechanisms based on the high frequency of incongruent trials could explain the non-significant results on the accuracy delta-plot in this condition. Moreover, if control is proactively maintained throughout the task, response inhibition mechanisms should already have some degree of activation (Ridderinkhof, 2002; West & Bailey, 2012) when easy-to-read incongruent trials are presented to the participant in this condition, hereby cancelling the fluency-driven responses that were observed in mostly congruent condition.

The delta-plot for RT is consistent with what has been previously reported in Stroop-like tasks (Pratte et al., 2010). We have found significant linear trends for easy and hard-to-read trials on the mostly congruent and mostly incongruent condition suggesting that interference increases over time on all conditions. However, we did not find evidence of difference in response inhibition to easy and hard-to-read trials as indicated by the absence of a significant difference on the last bin of the distribution and the slope connecting the last two bins (Wylie et al., 2009). Therefore, the effect of ease-of-processing in this experiment seems to be constrained to the response activation mechanisms already discussed.

In this experiment, we have also investigated the same control mechanism at a micro-level. We tested the effect of trial discrepancy within both proportion of congruence conditions through a pseudo-randomisation procedure that ensured that discrepant trials (incongruent on mostly congruent and congruent on mostly incongruent) would appear in single or repeated presentations, while balancing all transitions of perceptual fluency. Participants in the mostly incongruent condition, after experiencing the facilitation effect of repeated congruent trials (faster responses on the second trial), committed more errors in the following incongruent trial. If, as previously reported (Braver, 2012; De Pisapia & Braver, 2006; West & Bailey, 2012), control is sustained in the mostly incongruent condition and congruence signals that control mechanisms may relax (Berger et al., 2019), these results may be interpreted as a disruption of proactive control mechanisms, possibly by signals indicating that control is no longer required. Future research can explore this interpretation by showing also at a macro-level that proactive control is harder to be instantiated in contexts of high processing fluency. Moreover, current theories suggest that discrepancy itself entails an initial state of negative affect marking the unexpectedness of the stimulus, irrespective of its’ valence (Noordewier et al., 2016; Topolinski & Strack, 2015). This hypothesis provides an interesting perspective to our results. Although congruent stimuli are generally thought to elicit processing fluency and positive affect (e.g., Berger et al., 2019), in this specific context, there may be a time sensitive dynamic transition from an initial negative affect marking the discrepancy, to positive affect when the stimuli are evaluated as congruent at a later stage. Given the possible diagnostic use of affective cues by the monitoring system of control and the disruption we have found for proactive control, the cognitive processing of discrepant trials and their impact on control mechanisms requires further unravelling. Contrary to our predictions, we did not find an extensive effect for the transitions in stimulus legibility in these trial-by-trial adaptations. Instead, transitions to perceptual fluency only decreased the RT on congruent trials after experiencing incongruence in the mostly congruent condition. Despite the specificity of the effect, this result is consistent with our hypothesis that the effects of perceptual fluency and congruence are additive (Oliveira et al., 2022).

In conclusion, the results of this experiment highlight the efficiency of our control mechanisms and corroborate our view that special conditions must be created to mislead them. We only detect an enhanced maladaptive automatic response activation on easy-to-read trials, with increased errors in incongruent trials, when cognitive control is sufficiently relaxed. This deception is restricted to fast responses, since control mechanisms quickly adapt by increasing time spent within a trial. Moreover, we also provide evidence of the same mechanism occurring at the level of micro-adjustments. When control mechanisms are proactively triggered throughout the task, our data suggest that transient experiences of processing fluency associated with congruence are capable of sufficiently downregulating control mechanisms, leading to more errors on the following incongruent trial. Together with existing literature, our results lend support to the notion that the monitoring system of control may be using ease-of-processing as an affective cue indicating that control is not required. Consequently, incidental occurrences of ease-of-processing are likely to lead to impairments in cognitive control.

## Supplemental Material

sj-docx-1-qjp-10.1177_17470218231159787 – Supplemental material for Easy to process, hard to control: Transient and sustained processing fluency impairs cognitive control adjustments to conflict Click here for additional data file. Supplemental material, sj-docx-1-qjp-10.1177_17470218231159787 for Easy to process, hard to control: Transient and sustained processing fluency impairs cognitive control adjustments to conflict by Gonçalo A Oliveira, Miguel Remondes and Teresa Garcia-Marques in Quarterly Journal of Experimental Psychology
