# Supplementary material for: Easy to process, hard to control: Transient and sustained processing fluency impairs cognitive control adjustments to conflict
Source: Q J Exp Psychol (Hove). 2023 Mar 23;76(11):2524–34. doi: 10.1177/17470218231159787 (PMC10585938; doi:10.1177/17470218231159787)
Supplement: sj-docx-1-qjp-10.1177_17470218231159787 – Supplemental material for Easy to process, hard to control: Transient and sustained processing fluency impairs cognitive control adjustments to conflict [file sj-docx-1-qjp-10.1177_17470218231159787.docx]

Supplementary Material for:

**Easy to process, hard to control: transient and sustained processing fluency impairs cognitive control adjustments to conflict**

Gonçalo A. Oliveira^1,2*^, Miguel Remondes^2^, Teresa Garcia-Marques^1,3^

^1^ ISPA-William James Center for Research, Rua Jardim do Tabaco 34, 1100-304 Lisboa, Portugal.

^2^ Instituto de Medicina Molecular João Lobo Antunes, Universidade de Lisboa,
Avenida Professor Egas Moniz, 1649-028 Lisboa, Portugal.

^3^ ISPA-Instituto Universitário, Rua Jardim do Tabaco 34, 1100-304 Lisboa, Portugal.

*Corresponding author: [goliveira@ispa.pt](mailto:goliveira@ispa.pt)

**Table S1 - List of fonts used in easy-to-read and hard-to-read trials**

| **Easy-to-read trials** | **Hard-to-read trials** |
| --- | --- |
| Arial | Distortion Dos Analogue |
| Courier New | Cyberwhiz |
| Times New Roman | ExtraBlur |
| Calibri | Halcion |
| Cambria | Halcyon Days NF |
| Source Sans Pro | Horrendous |
| Century | Kandinsky |
| Malgun Gothic | Nervous |
| Verdana | Phantomime |
| Tahoma | MedicationNeeded |
| Trebuchet MS | Petroleum St |
| Bookman Old Style | Pulse State |
| Gentium Basic | Sans Forgetica |
| Bahnschrift Regular | SF Solar Sailer |
| PT Root UI | Snippletweak |
| Titillium WebThin | Son Of A Glitch |
| Qualio | This Corrosion |
| Glam Queen | Ko City |
| Garamond | Acidic |
| Liberation Sans | BinaryWaters |

**Table S2 - Fixed-effect statistics of the linear mixed models for the accuracy and reaction times delta-plots**

|  | Accuracy delta-plot | | |  |  | RT Delta-plot | | |
| --- | --- | --- | --- | --- | --- | --- | --- | --- |
| Effect | *df* | *F* | *p-value* |  |  | *Df* | *F* | *p-value* |
| Legibility | 1, 774 | .03 | .856 |  |  | 1, 84.88 | .53 | .466 |
| Bins | 4, 774 | 27.20 | <.001*** |  |  | 4, 676.01 | 44.54 | <.001*** |
| Proportion of congruence | 1, 86 | 15.98 | <.001*** |  |  | 1, 85.28 | 52.41 | <.001*** |
| Legibility x bins | 4, 774 | 1.91 | .105 |  |  | 4, 676.01 | .61 | .652 |
| Legibility x Proportion of congruence | 1, 774 | .52 | .467 |  |  | 1, 84.88 | .30 | .587 |
| bins x Proportion of congruence | 4, 774 | 16.13 | <.001*** |  |  | 4, 676.01 | 1.42 | .224 |
| Legibility x bins x Proportion of congruence | 4, 774 | 2.53 | .039* |  |  | 4, 676.01 | .16 | .956 |

df: degrees of freedom (numerator, denominator); *denotes a significant effect for p-value≤.05; *** denotes a significant effect for p-value≤.001

**Table S3 - Fixed-effect statistics of the linear mixed models for the target delta-plot slopes of accuracy and reaction times**

|  | Accuracy slope 2-1 | | |  |  | RT slope 5-4 | | |
| --- | --- | --- | --- | --- | --- | --- | --- | --- |
| Effect | *df* | *F* | *p-value* |  |  | *df* | *F* | *p-value* |
| Legibility | 1, 86 | 3.50 | .065 |  |  | 1, 85.1 | .30 | .585 |
| Proportion of congruence | 1, 86 | 20.70 | <.001*** |  |  | 1, 85.28 | .11 | .742 |
| Legibility x Proportion of congruence | 1, 86 | 4.76 | .032* |  |  | 1, 85.1 | <.01 | .979 |

df: degrees of freedom (numerator, denominator); *denotes a significant effect for p-value≤.05; *** denotes a significant effect for p-value≤.001

**Table S4 – Estimated marginal means for Accuracy and Reaction time of the discrepant trials**

| Proportion of congruence | Discrepant trial  presentation | Accuracy | | | | Reaction time (log10) | | | |
| --- | --- | --- | --- | --- | --- | --- | --- | --- | --- |
|  |  | EMM | SE | LCL | UCL | EMM | SE | LCL | UCL |
| MC | single | .934 | .013 | .904 | .956 | 2.883 | .012 | 2.860 | 2.906 |
|  | repeated | .936 | .013 | .906 | .957 | 2.897 | .013 | 2.872 | 2.922 |
| MI | single | .995 | .003 | .985 | .999 | 2.850 | .022 | 2.791 | 2.908 |
|  | repeated | .998 | .001 | .993 | 1.000 | 2.837 | .022 | 2.779 | 2.896 |

MC: Mostly congruent; MI: Mostly incongruent; EMM: Estimated marginal mean; SE: Standard error of the mean; LCI: Lower confidence interval; UCI: Upper confidence interval; Confidence intervals were calculated for 95%.

**Table S5 – Estimated marginal means for Accuracy and Reaction time of the following trial after exposure to discrepant trials**

| Proportion of congruence | Discrepant trial  presentation | Legibility  change | Accuracy | | | | Reaction time (log10) | | | |
| --- | --- | --- | --- | --- | --- | --- | --- | --- | --- | --- |
|  |  |  | EMM | SE | LCL | UCL | EMM | SE | LCL | UCL |
| MC | single | E-E | .991 | .005 | .976 | .997 | 2.822 | .023 | 2.767 | 2.876 |
|  |  | E-H | .993 | .004 | .979 | .998 | 2.845 | .023 | 2.790 | 2.900 |
|  |  | H-H | .995 | .003 | .982 | .999 | 2.844 | .023 | 2.789 | 2.899 |
|  |  | H-E | .995 | .003 | .982 | .999 | 2.817 | .023 | 2.762 | 2.872 |
|  | repeated | E-E | .988 | .006 | .970 | .995 | 2.824 | .023 | 2.769 | 2.878 |
|  |  | E-H | .990 | .005 | .973 | .996 | 2.833 | .023 | 2.778 | 2.888 |
|  |  | H-H | .993 | .004 | .979 | .998 | 2.840 | .023 | 2.785 | 2.895 |
|  |  | H-E | .988 | .006 | .970 | .995 | 2.830 | .023 | 2.776 | 2.885 |
| MI | single | E-E | .974 | .009 | .950 | .986 | 2.874 | .013 | 2.847 | 2.901 |
|  |  | E-H | .974 | .009 | .950 | .987 | 2.882 | .013 | 2.856 | 2.909 |
|  |  | H-H | .976 | .008 | .953 | .988 | 2.897 | .013 | 2.870 | 2.923 |
|  |  | H-E | .959 | .012 | .928 | .977 | 2.876 | .013 | 2.850 | 2.903 |
|  | repeated | E-E | .954 | .013 | .920 | .974 | 2.882 | .013 | 2.856 | 2.909 |
|  |  | E-H | .943 | .015 | .904 | .966 | 2.893 | .014 | 2.866 | 2.920 |
|  |  | H-H | .955 | .013 | .922 | .975 | 2.881 | .014 | 2.854 | 2.908 |
|  |  | H-E | .964 | .011 | .935 | .980 | 2.872 | .014 | 2.845 | 2.899 |

MC: Mostly congruent; MI: Mostly incongruent; E-E: both trials Easy-to-read; E-H: Easy-to-read to Hard-to-read; H-H: both trials are Hard-to-read; H-E: Hard-to-read to Easy-to-read; EMM: Estimated marginal mean; SE: Standard error of the mean; LCI: Lower confidence interval; UCI: Upper confidence interval; Confidence intervals were calculated for 95%;
